# An interdisciplinary team communication framework and its application to healthcare 'e-teams' systems design

**DOI:** 10.1186/1472-6947-9-43

**Published:** 2009-09-15

**Authors:** Craig E Kuziemsky, Elizabeth M Borycki, Mary Ellen Purkis, Fraser Black, Michael Boyle, Denise Cloutier-Fisher, Lee Ann Fox, Patricia MacKenzie, Ann Syme, Coby Tschanz, Wendy Wainwright, Helen Wong, Interprofessional Practices Team (alphabetically)

**Affiliations:** 1 Telfer School of Management, University of Ottawa, Ottawa, Ontario, Canada; 2 School of Health Information Science, University of Victoria, Victoria, British Columbia, Canada; 3 Victoria Hospice Society, Victoria, British Columbia, Canada; 4 British Columbia Cancer Agency, Victoria, British Columbia, Canada; 5 School of Nursing, University of Victoria, Victoria British Columbia, Canada; 6 School of Social Work, University of Victoria, Victoria, British Columbia, Canada; 7 School of Human Geography, University of Victoria, British Columbia, Canada

## Abstract

**Background:**

There are few studies that examine the processes that interdisciplinary teams engage in and how we can design health information systems (HIS) to support those team processes. This was an exploratory study with two purposes: (1) To develop a framework for interdisciplinary team communication based on structures, processes and outcomes that were identified as having occurred during weekly team meetings. (2) To use the framework to guide 'e-teams' HIS design to support interdisciplinary team meeting communication.

**Methods:**

An ethnographic approach was used to collect data on two interdisciplinary teams. Qualitative content analysis was used to analyze the data according to structures, processes and outcomes.

**Results:**

We present details for team meta-concepts of structures, processes and outcomes and the concepts and sub concepts within each meta-concept. We also provide an exploratory framework for interdisciplinary team communication and describe how the framework can guide HIS design to support 'e-teams'.

**Conclusion:**

The structures, processes and outcomes that describe interdisciplinary teams are complex and often occur in a non-linear fashion. Electronic data support, process facilitation and team video conferencing are three HIS tools that can enhance team function.

## Background

Interdisciplinary teams are an essential aspect of modern organizational work and are an important facilitator in achieving positive, cost-effective outcomes in various organizational settings [1]. Nowhere is interdisciplinary team communication more important than in health care settings as the complex nature and demands of the health care work environment requires the expertise and knowledge of differing individuals or specialists who can work together to solve multifaceted and complex patient care problems [2]. Research suggests that good interdisciplinary communication leads to improved patient and family outcomes (i.e. high levels of patient and family satisfaction, symptom control, reductions in length of stay and hospital costs) [3]. As well, research has demonstrated that interdisciplinary teamwork can improve the diagnostic and prognostic abilities of health professionals, more than individual health professionals working alone [3]. In recent years there have been significant advances in the development of technologies that support teamwork (e.g. groupware). However unlike other domains of practice where teams work on complex problems (e.g. engineering and computer science), team work in health care is more varied as a patient's medical condition(s) may vary in severity, complexity and uniqueness. Furthermore, team work in healthcare is often the norm and not the exception as there is a need to solve complex patient problems on a daily basis. From a technological perspective developing technologies that support such complex and unique work can be difficult.

The practice of patient care by interdisciplinary teams is particularly important in specialized health care settings such as palliative care. Current research suggests approximately 70% of deaths in North America are due to chronic illness [4]. Furthermore, it is expected that the number of individuals suffering from and living with chronic illness such as diabetes, heart disease and cancer will increase significantly over the next few years. These increases in the numbers of individuals living with chronic illnesses and the complexities associated with the long term management of chronic illnesses suggests there is a need for the expertise of interdisciplinary palliative care teams [4]. Interdisciplinary teams are considered an integral part of palliative care delivery [5]. Palliative care emphasizes quality of life and the relief of physical, psychosocial and spiritual suffering, which can only be achieved through interdisciplinary teamwork [6]. Few studies exist that have studied palliative care teams and how technology can support such teams. Demiris et al. [7] showed that information flow during interdisciplinary hospice team meetings can be deficient and limit the effectiveness of the meetings. They suggest interdisciplinary teams require a platform or infrastructure to guide the information sharing and communication that take place during team based care delivery.

To date health informatics research has tended to focus on representing and storing information despite the fact that up to 90% of information transactions in healthcare involve information exchange in order for communication to be successful [8]. In particular there are few studies that focus on how team communication occurs and how to design information and communication technologies to support basic communication processes [9]. Effective interdisciplinary team communication is essential for the prevention of medical errors [10,11]. Communication studies in healthcare include Reddy and Spence [12], who studied teamwork in the emergency department, and Alvarez and Coeira, who studied interruptive communication in the intensive care unit [13]. There is also extensive research on teamwork in other domains such as aviation [14]. However, there are two key shortcomings in the team communication research both inside and outside of healthcare. First, there is the need to understand and support communication practices across multiple settings. As Avison and Young point out, team processes such as decision making are more complex in healthcare because of the need to deliver highly integrated, personalized care via multidisciplinary teams located in differing settings (e.g. acute care hospital, community) [15]. The second shortcoming is that existing team communication research often provides broad recommendations such as "improve communication" without describing what type of communication actually occurs between health professionals or providing actionable guidance to improve communication [11]. For example, Saffran et al. [16] describes how health informatics applications can support team based tasks and collaboration, however, the study only looks at collaboration from a general perspective such as stating that collaboration is required for good patient care. Saffran et al. [16] did not examine team processes or patient cases within a specific context such as the palliative care environment nor did they discuss how they would design a HIS to support specific team structures, processes or outcomes. We argue that teams are complex entities and thus there is a need for research on specific healthcare team structures, processes and outcomes and how we can apply them to HIS design to support team practices across different settings.

This study addresses the two shortcomings of interdisciplinary team research outlined above, and presents results of an exploratory study on team communication structures, processes and outcomes. More specifically, the study aims are: (1) To develop a preliminary framework for interdisciplinary team communication that recognizes the structures, processes and outcomes that are employed by teams, (2) To use the framework to provide insight into HIS design to support interdisciplinary healthcare teams.

## Methods

### Data Sources

Our data sources consisted of two interdisciplinary palliative care teams located in two different institutions in an urban city in British Columbia, Canada. Team A was from an inpatient hospice unit whereas Team B was from an outpatient cancer agency. Team A was comprised of one physician, a physiotherapist, a team leader (who is a registered nurse (RN), a community case manager, a counselor, a spiritual care provider and a RN. Team B was comprised of three physicians, a team leader (who is an RN), a counselor, a pharmacist and a RN. In team B two of the physicians were palliative care specialists and the third physician was a medical oncologist. Team size varied slightly from week to week for both teams. Team A had between 5-8 members while team B had between 5-7 members depending on the meeting. All meetings for both teams were approximately 1 hour in length. Prior to commencing the study research ethics approval was obtained from the University of Victoria, Vancouver Island Health Authority and British Columbia Cancer Agency. All study participants provided written consent prior to data collection.

### Research Methods

We undertook a qualitative, ethnographic study of interdisciplinary team work. The ethnographic methodology was adapted from a video ethnography technique previously used in medicine to study patient consults [17], and from observational ethnographic techniques used to collect data in the intensive care ward [18] and emergency department [12]. The use of ethnography in health informatics studies differs from ethnographic studies in the social sciences where researchers become immersed in the culture of the participants being studied. Rather the use of ethnographic approaches in health informatics studies such as [17,18], and [19] involve the collection of data while observing participants in their natural settings. In our study we did spend time with the participants prior to data collection, which included discussing the study objectives with them and allowing them to see the recording equipment. This helped to establish a level of comfort with the participants with respect to having a video camera present in meetings, which was crucial for maintaining a natural environment.

Data was collected from the two teams in section 2 over a period of eight weeks. Both teams took part in team meetings once a week to exchange and communicate information about patient cases. The weekly meetings were observed, audio and video recorded by a researcher using recording methods developed in the field of health informatics [20,21]. The recording methodology involved audio and video recording of recording of team meetings using a Sony^® ^Mini DVD camera mounted on a tripod [20]. In addition to audio and video recordings of the team meetings, a trained qualitative researcher made observations and took field notes of team members communicating and interacting with one another to provide additional context for the data and to validate or triangulate the study findings. A total of ten meetings were recorded, four from team A and six from team B.

### Data Analysis

**Figure 1:**
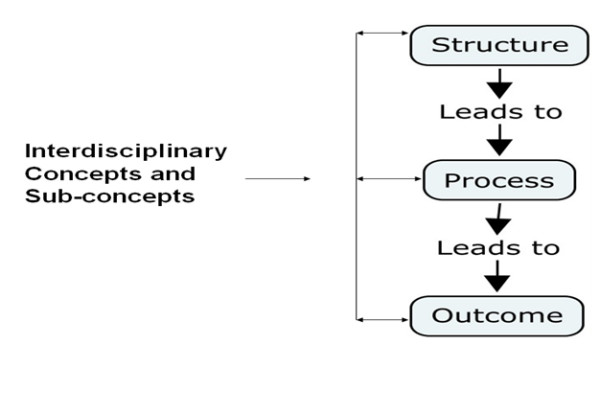
**Donabedian's framework with two way arrows illustrating connectivity between the three dimensions**.

Audio and video data were transcribed verbatim and analyzed using qualitative content analysis [22]. We used a directed qualitative analysis approach. The analysis was a hybrid data-theory driven approach. The data was initially coded without analysis through descriptive coding. A second coding cycle was then done where Donabedian's quality framework (i.e. structure, process and outcome) was used to guide the analysis (i.e. theory driven) [23]. After the data was analyzed using Donabedian's framework the data was coded again to identify emergent themes (i.e. concepts and sub-concepts using Donabedian's dimensions). It must be noted the emergence of concepts and sub concepts was non-linear as our data revealed teamwork did not follow a linear pattern. For example the data revealed that at times teams would start their work by focusing on patient outcomes and then attending to processes and structures that lead to those outcomes. Figure 1 shows Donabedian's framework with two way arrows illustrating cross concept emergence during team discussions, such as identifying an outcome that leads to a structure.

Our research team had a diverse, multidisciplinary background (e.g. health informatics, social work, medicine and nursing). That diversity provided a multidisciplinary approach when coding the data and led to greater attention to a range of issues such as how social dynamics impact teams, how the information needs of care providers (nurses, physiotherapists, counselors and physicians) differ, and how information is communicated amongst the differing team members. Each member of the research team received the audio transcripts and did their own data driven coding of the transcripts. The team would meet approximately once a week to watch the videos and discuss the coding. At the team meetings the codes were analyzed using Donabedian's dimensions. The videos enabled us to view non-verbal signals that were not available on transcripts, an example being team members scouring through various charting documents trying to find the relevant information for a patient case discussion. The group meetings also allowed us to reach group consensus when differences in coding existed. All the final concepts and sub-concepts that formed our framework were agreed upon by the research team.

## Results

Our findings are organized according to the meta-concepts of: structures, processes and outcomes. The concepts and sub concepts that emerged for each of the three meta-concepts were modeled as an ontology using an ontology development methodology [24]. We then describe each of the concepts and sub-concepts, using excerpts from the data to illustrate examples of the concepts and sub-concepts. We bold the text in some of the excerpts to draw attention to key points.

### Structures

**Figure 2:**
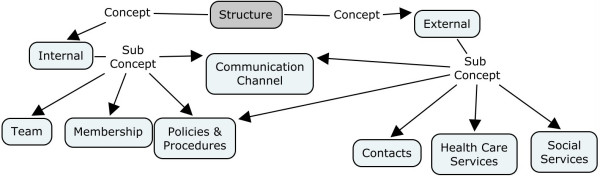
**Ontology of concepts and subconcepts for the structures of interdisciplinary team communication**.

In this study it was found that team structure had two main concepts, internal and external, with each of those concepts having a number of sub-concepts (See Figure 2). The communication channels sub-concept acted as a common concept that integrated internal and external team communication processes. Internal concepts determined how effectively the team functioned from within and included the membership, policies and procedures, and communication practices of the team members. External concepts influenced how teams coordinated their work with outside agencies and/or individuals.

Team awareness and implementation of the policies and procedures of both the internal and external structures was shown to be crucial for team success. Excerpt 1 is an example of effective communication as the team utilizes both the internal and external communication structures that affect the team. In the excerpt a patient is being seen by one of the interdisciplinary palliative care teams (Team B) but the patient also has an external physician, who is the primary physician for the patient. In the excerpt, Team B discusses and has questions about the patient's medication dose but Team B does not want to change the primary care physician's plan of care. Thus a physician from Team B requests that the team nurse communicate by telephone with the primary physician to inquire about how the primary physician is doing and to ask if he required any assistance. This illustrates the establishment of a communication channel between the internal and external structures that respects each structure's authority.

MD 1: But, I mean, if he's handed it back to [physician name], **then why are we getting involved without asking?**

RN 1: I'm not too sure.

MD 2: [Drug name] seems like quite a big dose as well.

MD 1: She, uh. There's no copied...stuff here, so we don't know who got a copy of this. **Why doesn't somebody phone [physician name] **and just say we've had this request from Dr. B and...

RN 1: Ok, I can do that.

MD 1: ...and does he want us to do anything about it? how is he managing? is he ok? because **I think otherwise, we are kind of meddling in his field**.

**Excerpt 1**: Example of external communication structure. (MD - physician, RN- registered nurse)

Communication structures are also influenced by interdisciplinary team communication in other ways. Teams preferred specific types of communication channels (i.e. telephone calls) over other types of communication. However, it is important to understand individual team member personal preferences with respect to communication channels. For example E-mail, although convenient, could not be used to communicate among team members as not all members of the team had access to email nor did all team members use e-mail with the same frequency. In the study data, an external physician e-mailed one of team B's physicians requesting that a patient's medication be changed. The team B physician acknowledged that a number of days passed before he read the message because he "does not check his e-mail that often". Asynchronous forms of communication such as e-mail may have disadvantages associated with their use as some team members may not use asynchronous channels of communication with the same frequency as others. Team agreement is needed in terms of: (a) the type of communication channel to be used, and (b) the frequency with which the communication channel should be monitored and used.

### Processes

**Figure 3:**
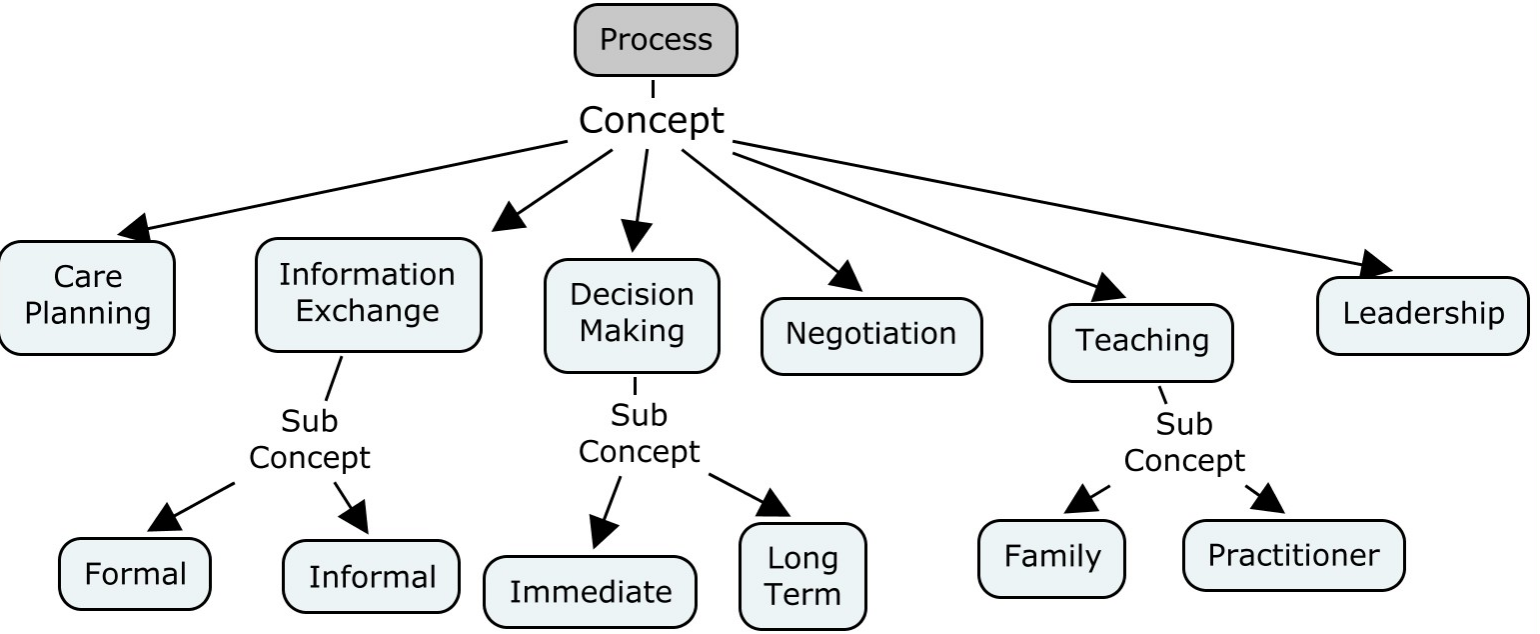
**Ontology of concepts and subconcepts for the processes of interdisciplinary team communication**.

We identified six team processes that were key aspects of communication: care planning, information exchange, teaching, decision making, negotiation, and leadership. Care planning, information exchange, decision making and teaching are primary processes while negotiation and leadership are supporting processes that take place in conjunction with other processes. Figure 3 shows the ontology of concepts and subconcepts for team processes. Each of the six team processes are defined and discussed below.

**Care Planning **is a common task in healthcare and refers to the need to ensure all aspects of patient care are addressed and followed. However care planning by interdisciplinary teams presents an added level of complexity as it requires input from both internal and external team members such as was illustrated in excerpt 1 above. Furthermore, care planning includes not only medical planning (such as orders for prescriptions or laboratory testing), but also non-medical planning such as coordination with social services. For example, one patient case in our data involved a physician coordinating with social services because a patient was unable to work because of her illness and the patient's government sponsored benefits were about to cease. The physician had to write a letter in order for the patient to receive financial and social support from the government because of her inability to work due to illness.

Part of care planning is coordinating the patient's care using multiple members of the care team. All of the patients from both teams A and B saw multiple health care providers and thus it was essential that the team lay out a plan of care that addressed the patient's health care needs as well as identified the roles and responsibilities of each healthcare provider in order to coordinate the patient's care. A key aspect of care coordination involves ensuring that all the roles and responsibilities of each team member are clearly defined so no team member assumes the roles and responsibilities of another team member, or that a task is not completed because the team members believe each other is responsible for completing the task.

**Information exchange **can be defined as the process by which team members interact with one another during team meetings. Information exchange can be formal and involve explicit or implicit forms of information technology. For example, formal exchange may involve discussing potential patient treatment options with other members of the interdisciplinary team. It may also be informal such as discussing organizational policies or other topics of interest with the team. In our study informal discussion usually took place before the team meeting or in-between discussions about patient cases. Team dynamics have been described as a valuable part of interdisciplinary teams [25] and we noted informal exchanges were supportive of interdisciplinary team dynamics. Team members who gathered together to participate in interdisciplinary team meetings often had informal discussions in order to exchange organizational information and to discuss organizational issues that might impact upon team activities aimed at managing patient care. These informal exchanges also allowed team members to discuss issues that were of concern to them. Excerpt 2 provides an example of this as a cancer oncologist (MD2) and a palliative care physician (MD1) talk during one team meeting about a drug research seminar they had both attended earlier that day and the amount of money spent on some anti-cancer drugs with limited evidence to support their use. The two physicians would likely not have such a discussion outside the team meeting with other staff but within the context of the team they felt comfortable discussing the issue.

MD2 - But it may explain why it doesn't work in some people. The [organization], it is fascinating the way it is spending millions, literally millions on some of these new drugs that are not available in other places.

MD1 - To what end?

MD2 - Good question. Some of the [type] drugs that came up this morning, we spend million's, I'm talking 2 or 3 million on some of these things, which we don't actually have hard survival data or good quality of life data on... sooner or later we are going to have to be accountable for, is this is a good use of our money, or could this money be used in other ways.

**Excerpt 2**. Information exchange. (MD2 - Cancer oncologist, MD1 - Palliative care physician)

**Team teaching, **which is where a team member provides teaching for the benefit of other team members or the patient and family, occurred frequently during interdisciplinary team meetings. Team teaching was used by team members to educate other members about patient health issues and provided additional disciplinary specific insight (e.g. nursing, pharmacy) into the patient case being discussed. Team teaching also helped team members to expand their palliative care knowledge base. To illustrate the effects of team teaching upon team processes we provide the following example. Three physicians were part of Team B - two were palliative care specialists while the third was a cancer oncologist. The cancer oncologist brought a unique perspective to the meetings - that of his knowledge of specific treatments such as chemotherapy and radiation. During one interdisciplinary palliative care team discussion about a patient, the oncologist described a phenomenon called 'chemo brain' where patients receiving chemotherapy may experience depression, memory loss or confusion. The palliative care physicians and other members of the team were not aware of 'chemo brain' as a post-treatment phenomenon and as a result were able to learn about the phenomena. Team teaching by the oncologist lead to the transfer of information about 'chemo brain' to other members of the palliative care team and more specifically the other palliative care physicians who were present at the meeting. After the 'chemo brain' concept was introduced the physicians discussed other patients who may have experienced 'chemo brain' as well as the need for data collection to monitor effects from chemo brain.

**Decision making **was the most commonly observed activity during meetings. However, the decision making process in interdisciplinary teams is complex as different team members are involved and there is sometimes a need to obtain input from both internal and external stakeholders before decisions can be made. One important precursor to team decision making was the ability of the team members to remain open to new ideas and to be willing to incorporate feedback from all team members into the team patient care plan. Excerpt 3 illustrates this. In this excerpt a physician describes a complex case and ends his description by saying he will be 'guided by what you think', referring to the interdisciplinary team. In excerpt 3 the physician clearly expresses his own perspective, but he acknowledges that that the complexity of the case requires further input and the advice of other team members.

MD- **I was going to suggest that she goes back to work just as a strategy **because she hasn't got...she's run out of [government social support] and I think even if she attempted to go back to work even though she doesn't want to... it would be a better thing than going back to work and relying on the inevitable things that are going to happen as a result of not going back to work such as eviction, food banks and whatever's going to happen there **so that was my thoughts on it but I'd be guided by what you think**...

**Excerpt 3**. A physician reaching out to team members for guidance and assistance (MD-Physician)

**Negotiation **is a supporting process that takes place as part of the decision-making process to achieve consensus about a decision as immediate consensus is not always reached in decision making. Although negotiation may begin within the context of a team meeting it often extends to activities outside the team meeting. For example, during a meeting of Team A it was determined that because of a patient's deteriorating condition, the patient and family would require homecare services to make it safe for the patient to be at home. However, the patient and family were very adamant that they would not accept such services. Therefore the team asked the physiotherapist member of the team to speak with the patient and family to negotiate a solution that would ensure the patient's return home was a safe one. Excerpt 4 illustrates this where a nurse (RN1) encourages the physiotherapist to work with the family to negotiate an acceptable solution. Excerpt 4 also shows a linkage between internal and external team structures because assuming the family is agreeable to accepting the homecare services the physiotherapist acknowledges that she would need to then contact the community case manager in order to make the necessary referral for services.

RN1 - if you could encourage her to...the case manager and the home care nurse are frustrated in trying to get a level of care in there that would be possible but with such resistance to it.....

RN2 (to PT) - are you going to do the community physio portion

PT - oh yes, I'm going to set that up....**I'm going to talk to her husband first, talk to them about equipment and see if they're willing to accept that physio may need to go in and**.... so provided the husband is okay with that I should contact the case manager and let them know the recommendations and let them deal with it from the community..they make the referrals...

**Excerpt 4**. Negotiation with a patient and family to accept care delivery (RN-registered nurse, PT-physiotherapist)

**Team leadership **also emerged from the transcript data as a supporting process to various processes, particularly decision making and teaching. Interdisciplinary team leaders played a key role in facilitating decision making and the exchange of information. In team meetings, where there was no clear team leader, the team often lapsed into random conversation, losing its focus (i.e. their focus drifted away from the patient being discussed). However, in cases where leadership was present, the team leader facilitated and focused team discussion upon the patient being discussed. For example, in Excerpt 5 a physician provides leadership to the team and helps the team to focus upon key patient care decisions.

MD - like I said before I think she is **quite a bit sicker than how she looks**, she looks good lying in bed but her electrolytes are all over the map...it could be that she could change quite quickly

Couns - I find her speech a little bit different too...just like she was having trouble finding her tongue....

RN - that's exactly what I noticed as well....

TL - so there **may not be a Christmas in Victoria**

MD - yeah that's it and I think we need to be **planning a little bit more of the immediate future rather than the distant future**

**Excerpt 5: **Leadership being used to focus the decision making process. (MD - physician, RN- registered nurse, Couns - counselor, TL - team leader)

In the discussion preceding excerpt 5 there had been discussion amongst the team about discharging the patient home that focused on understanding the patient's level of mobility and what types of arrangements would need to be made to support the patient in their home such as bathroom rails. The physician provided leadership to the team by focusing the discussion and pointing out that the patient was in the end stages of her disease process and that patient care planning should be focused upon the immediate future rather than the distant future. The physician indicated the patient was extremely ill and might not be able to return to her home. Therefore, the discussions about the patient's level of mobility and their need for health care supports were not appropriate given the patient's clinical state.

### Outcomes

**Figure 4:**
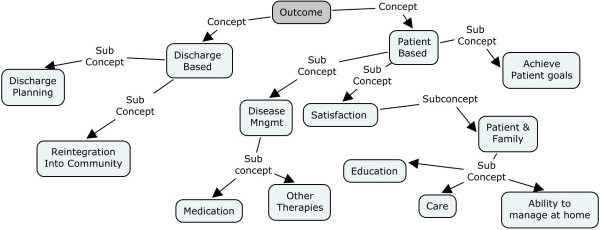
**Ontology of concepts and subconcepts for the outcomes of interdisciplinary team communication**.

We identified five team outcomes that were influenced by communication: patient discharge planning, the reintegration of the patient into community, effective disease management, patient and family satisfaction and patient achievement of goals and objectives. The outcomes were grouped into two categories: discharge based outcomes and patient based outcomes. Figure 4 provides the ontology of concepts and sub concepts for interdisciplinary team outcomes showing the grouping of the two aforementioned categories.

### Discharge Based Outcomes

Although patients are routinely discharged from hospitals or care centres, the discharge of patients receiving complex care such as palliative care requires significant coordination across the team members. As the two discharge outcome sub concepts illustrate, not only does discharge require communication across team members to ensure all requisite tasks are done to facilitate patient safety but it can also involve initiating external contacts to help patients reintegrate into their community.

**Discharge planning **was particularly important for the inpatient hospice patients in our study. Discharge planning of hospice patients involved significant teamwork that required much communication and coordination across a number of individuals from the team. The team had to attend to a number of patient care issues in order to effectively plan the safe discharge of the patient. Patient safety issues such as the need to ensure the right type of equipment was available to (e.g. guard rails, oxygen tanks, wheel chair ramps) was a common discharge discussion. Therefore, the physiotherapist's (PT) participation as a team member was essential. Excerpt 6 describes a PT coordinating a patient's discharge.

PT - basically I visited with her and did an assessment and she has a few things going on....**the home setup...they live...the main floor they have to go through a spiral staircase...so just so you know for the discharge we need someone to talk to them about [transportation home] **and for the...to bring her upstairs as that's where the main floor is and the kitchen, bathroom and bedrooms....

RN - up the spiral staircase?

PT - up the spiral staircase...it's a ground floor entry foyer and then the spiral staircase...so no it's not safe for her to do the stairs..you know up and down...she was quite dizzy and standing at the bedside for you know less than five minutes her sats went down to 89% air...so she is quite weak and she was doing more walking at home from what her daughter said and her falls might be due to that?I think her balance is precarious....

**Except 6 **- Physiotherapist coordinating discharge planning. (RN-registered nurse, PT-physiotherapist)

**Reintegration into the community **is a patient discharge outcome that attempts to help patients reestablish their lives in the community after disease treatment. Unlike discharge from surgery, where a patient may require follow up (e.g. physiotherapy) and then resume their life as it was before surgery, when patients are discharged from palliative care settings they often require team support to help them reestablish themselves in their community. Although much of team-based patient care is directed towards medical care activities such as diagnosing and treating disease, the team must address other aspects of patient care, particularly biopsychosocial care. Many of the patients seen by Team B (located in an outpatient agency) were treated and then discharged. Patients were expected to resume the lives they had before they became ill. However many of the patients, if not all, had their lives changed forever by their disease. Further, because of their illness some patients may not be able to resume their life as it was prior to their disease. For example, one patient being seen by Team B had been treated for cervical of cancer and was deemed "cancer free". However, the patient continued to suffer feelings of loss and experienced difficulty returning to work and family life. Excerpt 7 shows a discussion about issues the patient was having reintegrating into her community after her disease was treated.

Couns - I'm a bit concerned about the possibility of suicide....she had mentioned that when there was that fight with [husband name] she had thought about taking her life but she looked at her daughter and immediately thought I can't I've got kids but if physically she's cycling down and I also wondered about speaking to her GP about the possibility of anti-depressants because...I'm concerned about her and I think that's something we should keep a watch out for...

MD6 - that's my take on it because this is a serious thing..I mean she's now...they keep being threatened with eviction...it's almost that the situation is not solvable as he's paying maintenance for 2 other children ..for them...to get that to be reviewed requires them to spend some money which they don't have...

Couns - **what is seems to me she's experiencing is a lot of the losses and that's what's coming up is a lot of the survivorship issues and then with the upset**....the first marital spat is quite upsetting to anyone let alone someone who's quite sick and might be depressed...

**Excerpt 7**. Biopsychosocial issues impacting a patient's reintegration into the community. (MD - physician, Couns - counselor)

In the above excerpt, although the patient was "cancer free", the outcome to be achieved was not a medical outcome but rather one of aiding the patient to reintegrate into her community. Here, the team worked to coordinate with external agencies such as social services to help the patient access the necessary resources to be able to return to work and resume her role as a contributing member of the community. Some of the coordination that was done in this case included contacting government agencies to inquire about long term disability coverage for the patient, arranging payment plans to help the patient with financial debt, and having the team social worker facilitate counseling for the patient and her husband to try and overcome their family problems.

### Patient Based Outcomes

Patient based outcomes refer to the range of outcomes that are achieved as part of patient care. Patient outcomes include biomedical and psychosocial disease management as well as outcomes related to patient satisfaction with care and the achievement of patient goals and objectives.

**Disease management **refers to processes that are undertaken to manage chronic illness in order to slow the progression of disease or reduce the potential for future complications associated with the disease. Disease management reflects the interdisciplinary nature of the team and includes medical therapies such as drugs or invasive procedures but also includes therapeutic contributions from counselors or spiritual care providers who bring holistic and biopsychosocial perspectives to disease management. We observed that biopsychosocial or spiritual disease management can sometimes shed insight about a patient's physical disease status. Excerpt 8 describes a conversation a spiritual care provider had with a patient who was hiding the fact she had physical pain because of a fear of medications. The spiritual care provider has established trust with the patient and is using that trust to help the patient understand the need for pain management as part of quality of life.

SC - I had a nice spiritual visit with her yesterday... But she said an odd thing to me. I said to her, just out of the blue at some point, and she was moving, and I said, "are you hurting, are you in pain?" **And she said, "well I'm in pain, but I'm not telling them, they will just give me more drugs." **So I said, but you need something to help the pain. And you will have no quality of life if you are in pain all the time. So she has that thing, and a lot of people have that, "I have the pain, but don't give me any drugs to help me."

**Excerpt 8**. (SC - Spiritual Care provider)

**Satisfaction **is a multidimensional outcome. Part of patient satisfaction involves the interdisciplinary team educating the patient and their family about their care. Because of the biopsychosocial complexity that is associated with palliative care, interdisciplinary teams need to teach families how to self-manage disease processes. When patients are discharged home, informal caregivers (i.e. family) are responsible for much of the patient's care. As a result teams need to teach family members how to manage the patient's care once the patient is discharged home. The team must be satisfied that the informal caregivers have all the knowledge and skills that are needed to look after the patient after discharge. Excerpt 9 shows a discussion around a patient being discharged home just after having a catheter inserted. The community case manager (CM) and team leader (TL) is ensuring that all the required teaching around care of the catheter is done and that the family is aware of their responsibilities.

CM - Is just that the patient requires a catheter and home nursing will not do that and **unless the family are going to do all of the catheter care**

RN - I'm sorry I wasn't aware of that problem

CM - **you're going to do the teaching on the ward?**

MD - but the daughter is going home isn't she?

RN - yes that's why she wanted the trial now, while she's still here

TL - well if you wait for the 24 hours to see if she keeps it then that's Wednesday, so Wednesday evening you're going to have to have some kind of discussion with the husband about how to empty it, wash around it, all of that...can she wash herself?

**Excerpt 9**. Team coordinating teaching and other discharge activities to be satisfied with a patient and families ability to manage at home. (CM- Case manager, MD - physician, RN- registered nurse, Couns - counselor, TL - team leader)

**Achievement of patient goals and objectives **is perhaps the most important outcome that interdisciplinary teamwork. However, achieving patient goals and objectives can be difficult due to the changing biopsychosocial nature of disease. Sometimes it is necessary to draw upon other processes such as negotiation to achieve patient goals and objectives. Excerpt 10 provides an example of a patient whose goal was to be home for Christmas. Although the team believed the patient would certainly survive the six weeks until Christmas they had doubts the patient would be able to manage a discharge of two or three days as the patient was frail. In excerpt 10 the nurse states she has spoke to the patient and attempted to obtain a better understanding of the patient's hopes and wishes and how they might be achieved given the patient's disease progression.

RN- **but if the dream goal was to be home for Christmas I asked her what would that mean?, **would that be Christmas day, a couple of days?, boxing day would be the big day, that's the big [type] of holiday for her, so we talked about how the big dream would be to be home for a couple of days and the boys would be here and **perhaps we could adjust that dream given where her condition may be in that time**... whether that dream shrunk to a day pass or shrunk to dinner here. She was really quite open to having those discussions and accepting the change in condition whatever that may look like.

**Excerpt 10**. RN discussing communication process with a patient to understand a patient's (RN- Registered nurse)

In having an open and honest discussion with the patient the nurse was able to identify the patient's hopes and wishes (i.e. to spend time with her family over the holidays). It was also made clear, that although the patient wanted to be at home, her real goal was to have her two sons with her at Christmas.

### Interdisciplinary Team Communication Framework

**Figure 5:**
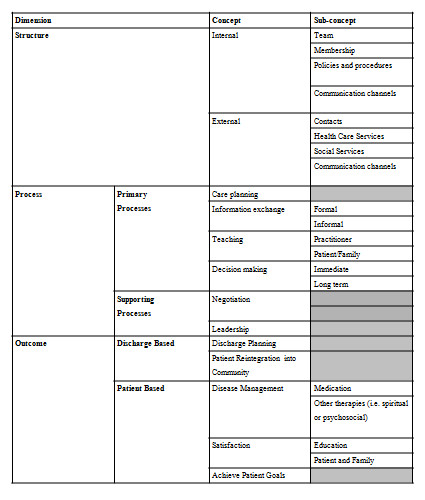
**Interdisciplinary team communication framework**.

Figure 5 provides a summary of the results in the form of an interdisciplinary team communication framework based on the three meta-concepts from Donabedian (structure, process and outcome). The framework can be used for assessing interdisciplinary teams by drawing attention to the range of factors that need to be considered for interdisciplinary teamwork and considering those factors in the context of the specific examples we have provided in this paper. Not all concepts have sub-concepts and such instances are portrayed with a gray box.

### Implications for 'e-Teams' Systems Design

**Figure 6:**
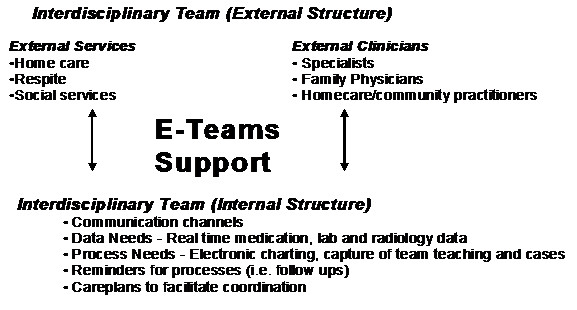
**e-teams support to facilitate interdisciplinary team communication between both internal and external structures**.

We see two key issues from the results we have presented in this paper. First is coordinating the negotiation of interdisciplinary team healthcare delivery and second is organizing and mediating the coordination process itself. Drawing upon the framework presented in the previous section we have developed a model for health information systems (HIS) design to support 'e-teams'. Figure 6 shows the 'e-teams' model and how it is based on the need to provide electronic support for both the internal and external structures of the team as well as the processes that take place within those structures. The e-teams model is a means of connecting the internal and external structures to a process facilitation tool (e.g. videoconferencing, electronic patient record) in order to enhance team outcomes. The e-teams model is a preliminary model that is meant to provide insights as to the opportunities and challenges of designing a HIS to support interdisciplinary teams for palliative care delivery.

We have identified three specific types of e-team supports necessary for effective team practices. The three supports are: data support, process facilitation, and video or web conferencing. Drawing upon the results from this paper we briefly discuss each type of e-team support and the challenges of implementing each support. Although research exists on each of those three types of support the literature has not specifically explored them in the context of interdisciplinary palliative care teams. Further we emphasize that solving the complexities of interdisciplinary based care requires more than just technological solutions. Rather the work processes that teams engage in must be understood and coordinated in the context of the technology being used.

### Electronic Data Support

Accurate up-to-date data is crucial for decision making. However in complex interdisciplinary care the access and sharing of data can be problematic because there is often for multiple care providers. The patient's medical records, which are largely paper based, may be transferred across different settings and updated frequently within those different settings. Therefore, when the interdisciplinary team meets they may be making decisions with inaccurate or incomplete data. Indeed our analysis showed several examples where team members would question whether data such as a medication dose was accurate or whether a patient had been seen by a specialist they were referred to. Both Team A and B spent precious meeting time searching for data and questioning whether data was accurate, which took away from time spent on actual team processes outlined earlier in the data analysis section. That raises issues about patient safety and the efficient use of team resources.

Because a palliative patient's medical record may be used and updated across differing settings an electronic record would be an ideal solution as it could provide real time data to the team including data on medications, laboratory tests, radiology results and external agencies such as social services or home care services. An electronic patient record would allow health professionals in differing settings to update the patient's data while allowing other sites to have real time access to the updated data. However, a single setting electronic patient record (EPR) is impractical for interdisciplinary team collaboration because as shown in this study an EPR system needs to link internal and external team structures. For example a patient in Team B may be in an EPR system at the cancer centre but such data would not be available to primary care physicians in the community. Similarly, patient data from a homecare system in the community may not be shareable with a cancer centre EPR. A move towards web-based EPR systems that offer secure access to data over the Internet is one solution to that problem. A further challenge in data support is that EPR or electronic data systems typically do not contain psychosocial or other palliative care relevant data elements [26]. In the patient based outcomes section an example was provided of how spiritual care played a key role in understanding a patient's denial of physical pain because of fear of opioids. If that data was not charted other providers would not be aware of the patient's fear of opioids and would be missing key data about the patient. That could result in discussions about the patient case being more medically focused because that data is readily available. Other studies have also described the value of spiritual care as part of interdisciplinary collaboration and the need for improvements to the chaplain's role within the interdisciplinary team process [27]. Studies have also shown that it is more normative for teams to share biomedical information as opposed to psychosocial information, which makes communication in team meetings more biased towards biomedical factors [28]. Enhanced biopsychosocial data collection is one way of strengthening the role of spiritual and psychosocial care.

### Electronic Process Facilitation

In this study both Team A and Team B had limited time for patient discussions. However, we observed numerous examples of time not being used efficiently by team members such as searching for medication data or test results or clarifying whether a task had been completed or not.

This study identified specific team processes (Figure 3) that could be enhanced through electronic support. Specifically, care planning and team teaching are examples of processes that can facilitated through the use of a health information system (i.e. electronic record). In our results we described the process of team teaching and how it makes a valuable contribution to team meetings by drawing on the differing expertise of team members. Decisions made by interdisciplinary palliative care teams are complex and incorporate experience, opinion and ethics. In the team teaching section we described how a medical oncologist introduced a concept called chemo brain that was used by the team as part of a patient case. However, because the team's composition is dynamic (i.e. health professional membership may change from week to week and individual team members may change), team members and the expertise they bring with them is not static. Ideally, it would be valuable to capture team teaching electronically to develop a knowledge base of team teachings that would provide knowledge that could be used in future team meetings. Such knowledge could be used as a form of decision support or as an educational tool by teams during weekly patient care meetings.

Another process that could be enhanced electronically is care planning. Currently all the patient documentation for teams A and B is paper based. Given the complexity of palliative care cases, the amount of data (e.g. medications, laboratory tests, radiology results and patient histories), and the number of decisions required for each patient case, an electronic care plan could ensure that all requisite processes are both initiated and completed. For example in excerpt 2 the physician points out that the patient is more ill than she appears and team decision making should be about the immediate future rather than the distant future. Such information would be noted in an electronic record (i.e. in the form of a care plan) to ensure that any subsequent decisions are made within a short time frame. Furthermore, as outlined in the description of the care planning process section of this paper it is important that tasks be assigned to a team member to ensure task completion and that there is no overlap by team members. Existing tools like electronic checklists and reminders of pending tasks would enhance task completion. However a challenge is that team tasks need to be coordinated in order to use such tools to their full advantage. In the structures section we described how a physician e-mailed a second physician with a medication change request and the message waited in his inbox for a week because the latter physician did not regularly check his e-mail. Ash, Berg and Coeira [29] describe a difference between information transfer and communication by pointing out how a computer order entry system was a barrier to an existing communication practice. It cannot be assumed that a HIS will automatically enhance processes like communication or decision making but rather the use of a HIS to support specific processes must be coordinated and agreed upon by all team members.

### Video or Web Conferencing

Video or web conferencing could be used to have team discussions about patient care when team members cannot physically attend a meeting. Clinicians from both teams in our study described how it would be much more efficient if they had real time communication with external team members, such as primary care physicians. There were numerous examples in the data of team members mentioning that they found it difficult to coordinate patient care from a distance. In excerpt 1 Team B is deciding the extent to which they should become involved in the patient's case because the patient already has a primary care provider. Team B does not want to 'meddle' in another physician's medical planning. In excerpt 1 Team B used additional meeting time to discuss what to do with the patient, but then a physician from team B has requested a nurse phone the primary physician to come to an understanding about how best to provide care for the patient. Video or web conferencing would enable providers external to the interdisciplinary teams to participate in meetings in real time so that decisions could be made quickly and to reduce delays associated with information exchange and coordination. Videoconferencing has been shown to be a practical means of palliative consults [30]. However much of the existing research has been on synchronous one-to-one consults. A challenge to video or web conferencing for interdisciplinary meetings with several providers across different settings is that it would require coordination between the internal and external team members, such as setting a meeting time and agenda. An external provider (i.e. family physician) will not want to sit through the entire team meeting but rather will only want to participate in the part of the meeting relevant to their patient. The team meetings are currently not scheduled with time slots for each patient but rather the patient discussions evolve through the course of the meeting. However time based coordination would need to be considered if multiple providers were to attend the meeting via electronic means. A benefit of video or web conferencing is that there would be less back and forth communication between team members (i.e. as in phone calls), which could facilitate better coordinated and more efficient patient care.

## Discussion

This study provided a methodological approach for studying interdisciplinary teams, a framework for analyzing team structure, processes and outcomes, and a model for e-teams system design. We extended existing research on interdisciplinary teams by using complex patient cases to define specific processes and outcomes as part of care delivery. As well, we were able to illustrate the relationship between internal and external team structures as well as the need to integrate them. We also discussed how the structures, processes and outcomes from our findings could be used to inform the design of a HIS to support e-teams.

We illustrated the non-linear fashion of team structures, processes and outcomes and how it is sometimes necessary to start with a desired outcome and reverse engineer to ensure the required structures and processes are in place to achieve that outcome. For example one of the team outcomes was satisfaction, which had the sub-concepts of: "patient and family", "education" and "ability to manage at home". Most of the patients from Team A were discharged home at some point and thus Team A had to coordinate the discharge plans through teamwork to ensure patient satisfaction. As a patients' family is largely responsible for providing care at home both the patient and family need to be satisfied with the discharge planning process to successfully care for the patient at home. Achieving patient and family satisfaction as a team outcome requires that teams coordinate discharge planning processes such as teaching the family how to move the patient (e.g. up and down stairs or turning in bed) and how to care for devices such as catheters or oxygen tanks that are needed to support the patient. Those teaching processes require a specific team structure that included nurses, physiotherapists and physicians.

We have also illustrated the complexity of communication in interdisciplinary settings. Although communication is often used as a general term there are times when it in fact means something more specific. For example communication can serve as a social fabric across both internal and external team members. We illustrated that fabric through team B's respect for an external physician's authority and their desire not to overstep their bounds. In that example team B used internal communication to establish their boundary and then used external communication to ask the external physician if he required any assistance. That social fabric is crucial for developing and maintaining trust across different team members and we must ensure we consider social fabric as we automate team processes.

The results from this paper have made contributions to the research and design of HIS to support team practices. From a research perspective we have further illustrated the value of qualitative based methods such as ethnography and content analysis for understanding the intricate processes that takes place during healthcare delivery. Team processes that we observed and subsequently included in our interdisciplinary team communication framework such as team teaching and information exchange might be viewed as informal processes as compared to decision making yet the informal processes were an important part of team functioning and dynamics. Using an ethnographic approach allowed us to observe teams in their natural setting and the videoethnographic technique produced an electronic record to enable our diverse research team to be able to contribute to the data analysis.

From a systems design perspective we discussed how electronic data support, electronic process facilitation and video or web conferencing could be used to support 'e-teams'. The e-teams model is different from standard hospital or web based EPR systems in that it is designed to support specific team structures and processes such as web or videoconferencing to connect internal and external team structures, facilitation of team processes such as teaching and care planning, and providing reminders and alerts to ensure completion of team tasks. The e-teams model could also enhance health outcomes in a measurable way. Although interdisciplinary teams are advocated as improving patient and family outcomes there are few studies that provide empirical evidence to support that claim. By collecting data on team based processes and outcomes we would be able to analyze metrics such as resource utilization by teams and patient and family satisfaction with team based care delivery. Finally we emphasize that HIS design to support e-teams is not just applying technological tools into team meetings but rather it requires an understanding of the specific processes teams engage in and the coordination needed to support those processes.

Limitations of our study include the fact it was a preliminary study that only studied two teams, which may limit the generalizability of the results. The framework we developed needs to be validated and studied in the context of other team based settings. We also focused our study on the team meetings and did not observe the completion of tasks by individual team members following the meetings. Our rationale was that team meetings act as the starting point for interdisciplinary team activities and is the place where team member tasks are identified. Future research will need to explore the relationship between the team meetings and the completion of team tasks by individual members by following patient cases longitudinally over time. A further limitation is that the paper used palliative care as the domain area to study interdisciplinary team structures, processes and outcomes. However the team communication framework and e-teams systems model provides a starting point for further research of teams in palliative care as well as in other domains of healthcare (e.g. ICU, pediatrics) and other industries such as production management or engineering. Future work will entail seeing the extent the findings from this study transfer to other settings and fully developing and testing the e-teams model in different interdisciplinary team settings.

## Conclusion

The practice of interdisciplinary teamwork is particularly important in specialized health care settings such as palliative care. The types of patient care problems that palliative care teams encounter are both complex and enduring, which makes them an ideal circumstance to study teamwork. In this paper we identified interdisciplinary team structures, processes and outcomes that emerged during our study of weekly team meetings. We then presented an interdisciplinary team communication framework and discussed HIS design to support e-teams.

## Competing interests

The authors declare that they have no competing interests.

## Authors' contributions

All authors were involved in the development of the study design and initial review of the data. CEK, EMB and MEP performed subsequent data analysis and initial drafting of the manuscript. CEK and EMB revised the manuscript. All authors approved the final version of the manuscript.

## Pre-publication history

The pre-publication history for this paper can be accessed here:

http://www.biomedcentral.com/1472-6947/9/43/prepub
